# Lebensqualität, Krankheitslast und Versorgungsbedarf von Patienten mit Vitiligo

**DOI:** 10.1007/s00105-024-05312-z

**Published:** 2024-03-11

**Authors:** Matthias Augustin, Caroline Gewiss, Nesrine Ben-Anaya, Markus Böhm

**Affiliations:** 1 Institut für Versorgungsforschung in der Dermatologie und bei Pflegeberufen (IVDP), Universitätsklinikum Hamburg-Eppendorf (UKE), Martinistr. 42, 20246 Hamburg, Deutschland; 2 Klinik für Hautkrankheiten, Universitätsklinikum Münster, Münster, Deutschland

**Keywords:** Psychosoziale Belastung, Autoimmunerkrankung, Depressionen, Pigmentverlust, Selbsthilfegruppe, Psychosocial burden, Autoimmune diseases, Depression, Hypopigmintation, Self-help groups

## Abstract

**Hintergrund:**

Vitiligo ist mit einer Prävalenz von 0,5–2,0 % eine der weltweit häufigsten Hauterkrankungen mit einem Verlust des Pigments. Die Hauterkrankung hat einen entstellenden, oft stigmatisierenden Charakter und ist oft mit psychosozialen Belastungen assoziiert.

**Zielsetzung:**

Es erfolgt eine Übersicht über die psychosoziale Beeinträchtigung, Krankheitslast und den resultierenden Versorgungsbedarf von Patienten mit Vitiligo.

**Material und Methoden:**

Es handelt sich um eine narrative Übersichtsarbeit auf der Grundlage einer Literaturrecherche in PubMed für die Jahre 1996 bis 2022 zu den Themen Krankheitslast, Lebensqualität und Stigmatisierung.

**Ergebnisse:**

Die Recherche ergab für den Suchzeitraum 175 relevante Originalarbeiten inklusive klinischer Studien, Metaanalysen und systematischer Übersichtsarbeiten (*n* = 65). Dabei dokumentiert eine Vielzahl an Studien, dass Vitiligo bei den Betroffenen mit erheblichen psychosozialen Belastungen und relevanten Einbußen an Lebensqualität einhergeht. Problembereiche betreffen insbesondere Stigmatisierung, sexuelle Funktionsstörungen, Angst, vermindertes Selbstwertgefühl und Probleme im Beruf. Das beobachtete erhöhte Ausmaß von Angst und Depressionen korreliert mit der Schwere und Aktivität der Vitiligo. Oft trägt auch Komorbidität zur weiteren Krankheitslast bei. Diese Faktoren determinieren bei einem relevanten Teil der Betroffenen einen hohen Versorgungsbedarf.

**Diskussion:**

Die Vitiligo stellt nicht in erster Linie ein kosmetisches Problem dar, sondern eine behandlungsbedürftige Erkrankung im Sinne der Definition der Weltgesundheitsorganisation von Gesundheit als körperliches, geistiges und soziales Wohlbefinden. Die Nutzen von Behandlungsoptionen sind an ihren Effekten auf patientenberichtete Endpunkte zu messen.

**Graphic abstract:**

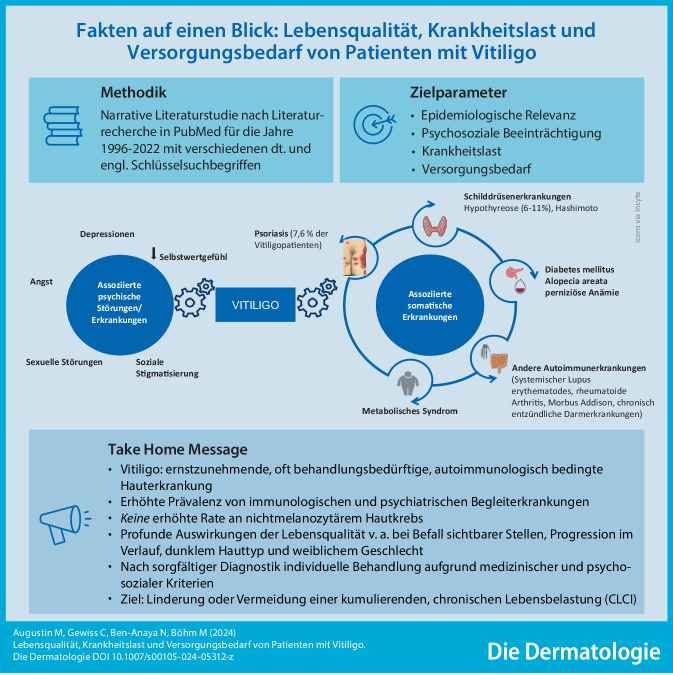

## Abstract (en)

**Background:**

With a prevalence of 0.5–2%, vitiligo is one of the most common skin disorders worldwide with loss of pigment. The skin disease has a disfiguring, often stigmatising character and is often associated with psychosocial distress.

**Objective:**

To provide an overview of the psychosocial impairment, disease burden and resulting health care needs of patients with vitiligo.

**Materials and methods:**

Narrative review based on a literature search in PubMed for the years 1996–2022 on disease burden, quality of life and stigmatization is provided.

**Results:**

The search yielded 175 relevant original papers including clinical studies, meta-analyses and systematic reviews (*n* = 65) for the search period. A large number of studies document that vitiligo is associated with considerable psychosocial stress and relevant losses in quality of life. Problem areas particularly concern stigmatisation, sexual dysfunction, anxiety, reduced self-esteem and problems at work. The observed increased levels of anxiety and depression correlate with the severity and activity of vitiligo. Often, comorbidity also contributes to reduced self-esteem and social isolation. These factors determine a high need for care in a relevant proportion of those affected.

**Conclusion:**

Vitiligo is not primarily a cosmetic problem, but a disease requiring treatment according to the World Health Organisation’s definition of health as physical, mental and social well-being. The benefits of treatment options are to be measured by their effects on patient-reported outcomes.

Vitiligo ist eine häufige Erkrankung der Haut, die oft mit einem chronischen Pigmentverlust einhergeht. Die weltweite Prävalenz ist hoch. Bei einem relevanten Teil der Betroffenen handelt es sich um eine ernst zu nehmende Erkrankung mit einem entstellenden, stigmatisierenden Charakter. Entsprechend ist Vitiligo häufig mit psychosozialen Effekten assoziiert und kann die Lebensqualität (LQ) der Patientinnen und Patienten erheblich beeinträchtigen. Aufgrund des hohen Leidensdrucks in Abhängigkeit von Ausmaß und Lokalisation der Pigmentstörung ist bei vielen Betroffenen eine spezifische Behandlung notwendig.

Vitiligo ist eine Erkrankung der Haut, bei der es zu einem Pigmentierungsverlust mit nachfolgenden depigmentierten Flecken kommt [28]. Diese sind meist umschrieben, können aber auch großflächig vorkommen und alle Hautregionen einnehmen. Klinisch wird Vitiligo in den segmentalen und den nichtsegmentalen Subtyp klassifiziert. Die segmentale Vitiligo tritt auf einer Körperhälfte auf und zeigt eine segmental bandartige Verteilung, während die nichtsegmentale Vitiligo typischerweise beidseits an Akren und Gesicht oder symmetrisch am ganzen Körper auftritt. Häufig sind die Streckseiten betroffen. Das Durchschnittsalter bei Beginn der segmentalen Vitiligo betrug in einer südkoreanischen Studie an über 200 Patienten 15,6 Jahre [38]. Die autoimmun vermittelte, nichtsegmentale Vitiligo zeigt eine bimodale Verteilungskurve im Beginn der Erkrankung. Ein früher Peak liegt bei einem Durchschnittsalter von 10,3 Jahren und ein später im Durchschnittsalter von 34,0 Jahren [41].

Pathogenetisch beruht der Pigmentierungsverlust nach heutigem Verständnis auf einer autoreaktiven, d. h. von körpereigenen Immunzellen vermittelten Schädigung der pigmentbildenden Melanozyten. Für die autoimmunologische Genese spricht auch die Assoziation von Vitiligo mit einer Reihe anderer autoinflammatorischer Erkrankungen, wie z. B. autoimmunen Schilddrüsenerkrankungen, rheumatoider Arthritis, Psoriasis, Diabetes mellitus oder Morbus Addison [11, 35]. Man geht heute davon aus, dass ca. 80 % des Vitiligorisikos auf genetische und ca. 20 % auf Umweltfaktoren zurückzuführen sind [10]. Im Rahmen genetischer Untersuchungen wurden mehr als 50 Genloci identifiziert, die die individuelle Suszeptibilität vermitteln, an Vitiligo zu erkranken [11]. Dazu zählt ein Bereich des Gens, das für Tyrosinase codiert, ein Enzym, das den geschwindigkeitslimitierenden Schritt der Melaninbiosynthese bildet [56] und ein wesentliches Antigen bei der generalisierten Vitiligo darstellt [7, 43, 60]. Bemerkenswert ist, dass mehr als die Hälfte der 50 Gene, die bei Vitiligo möglicherweise verändert sind, bei der Immunregulation beteiligt sind. Dies erklärt die Neigung von Patienten mit nichtsegmentaler Vitiligo, auch an anderen Autoimmunerkrankungen zu erkranken. Genomweite Assoziationsstudien an einer weltweiten Kohorte von Patienten mit nichtsegmentaler Vitiligo konnten zuletzt 23 neue Empfänglichkeitsgene identifizieren, die wichtige Signalwege unter anderem in der Immunregulierung und Apoptose kontrollieren [40].

Bei vielen Betroffenen ist aufgrund des hohen, krankheitsbedingten Leidensdrucks eine Behandlung erforderlich. Für diese stehen bereits Arzneimittel und andere Therapien zur Verfügung [9, 31], die dazu beitragen, den Krankheitsprozess aufzuhalten, depigmentierte Läsionen zu stabilisieren oder die Repigmentierung zu stimulieren [32, 61]. Allerdings ist deren therapeutischer Nutzen bisher begrenzt [6]. Darüber hinaus befinden sich neuere systemische und topische Wirkstoffe in der klinischen Erprobung, die noch zielgenauer in die Pathogenese der Vitiligo eingreifen [36, 42].

## Epidemiologische Relevanz von Vitiligo

Vitiligo zählt zu den weltweit häufigsten Pigmentierungsstörungen [26]. Ihre Prävalenz wird mit 0,5–2 % angegeben [30]; es gibt jedoch deutliche geografische Unterschiede. So weisen die Studiendaten auf einen höheren Leidensdruck in Ländern mit einem höheren Anteil an dunkelhäutiger Bevölkerung hin. Das klinische Problem der Vitiligo wird insgesamt wahrscheinlich unterschätzt, da sich Patienten aufgrund ihres sozialen Hintergrunds oder ihrer Bildung oder aufgrund mangelnder effektiver Therapieoptionen nicht in der dermatologischen Sprechstunde vorstellen [49, 57]. Die tatsächliche Prävalenz der Vitiligo unter Einbeziehung nicht diagnostizierter Fälle ist daher eher höher anzusetzen. Einer Umfrage mit 35.694 Teilnehmern zufolge liegt die Prävalenz in Europa, USA und Japan bei ca. 1,3 % der erwachsenen Bevölkerung [12]. Die weltweit höchste Prävalenz wurde mit 8,8 % in Indien angegeben, wobei im Rahmen der Erhebung auch Fälle von chemisch induzierter Depigmentierung erfasst worden waren [9].

Zur Epidemiologie in Deutschland liegen die Daten einer vom Institut für Versorgungsforschung in der Dermatologie und bei Pflegeberufen (IVDP) untersuchten Kohorte von Werktätigen (*n* = 121.783; 57 % männlich; Durchschnittsalter 43 Jahre) vor. In dieser Untersuchung betrug die Prävalenz der Vitiligo 0,77 % (0,84 % bei Männern; 0,67 % bei Frauen) [49]. In einer Analyse von Sekundärdaten der gesetzlichen Krankenversicherung (*n* = 1.619.678; 38 % männlich; Durchschnittsalter 46 Jahre) lag die (Behandlungs‑)Prävalenz bei 0,17 % (0,14 % bei Männern; 0,18 % bei Frauen). Diese Diskrepanz deutet darauf hin, dass sich ein relevanter Teil der Personen mit Vitiligo nicht (mehr) in ärztlicher Behandlung befindet. Eine Erklärung dafür könnte die aus Sicht der Patienten geringen Nutzen der bisherigen Therapien sein [5]. Vitiligo war dabei signifikant häufiger bei Personen mit hellem Hauttyp, Epheliden und kongenitalen Nävi sowie mit einer Vielzahl weiterer Hauterkrankungen verbunden [49]. In einer weiteren vom CVderm untersuchten bundesweiten Kohorte trat Vitiligo unter 1023 betroffenen Personen am häufigsten an den Händen (92 %), im Gesicht (87 %) und im Genitalbereich (86 %) und somit an besonders sensitiven, sichtbaren oder schambesetzten Arealen auf [59]. Ein weiterer Grund für die fehlende Inanspruchnahme könnte auch eine Bagatellisierung von Vitiligo innerhalb der Ärzteschaft sein.

## Psychosoziale Belastung und Lebensqualität

Die Haut ist für die Interaktion des Menschen mit seiner Umwelt essenziell. Vor allem sichtbare Hautveränderungen wirken sich auf das psychosoziale Befinden aus und bedeuten für die Betroffenen oft eine Stigmatisierung. Eine Vielzahl deutscher [58, 68] und internationaler [16, 22, 26, 37, 45, 51, 63, 69, 74] Studien hat gezeigt, dass Vitiligo mit einer erheblichen psychosozialen Belastung und einer relevanten Einbuße an LQ einhergeht. Zur Messung der LQ von Patienten mit Vitiligo eignen sich sowohl generische Instrumente wie der Short Form 12 (SF-12) als auch krankheitsspezifische Skalen wie die (Family) Vitiligo Impact Scale (VIS) [2], das Vitiligo-Specific Quality-of-Life Instrument (VitiQoL) [47] oder die Vitiligo Impact Patient Scale (VIPs) [64]. Der Dermatology Life Quality Index (DLQI) ist hingegen nur teilweise geeignet [59], da ein Teil der Items nicht regelhaft zutrifft.

Im Rahmen einer Literaturrecherche in PubMed für die Jahre 1996 bis 2022 zu den Themen Vitiligo und Krankheitslast, LQ oder Stigmatisierung fanden sich in 175 Originalarbeiten, davon 65 klinische Studien, Metaanalysen oder systematische Übersichtsarbeiten, eine gegenüber nicht betroffenen Menschen signifikant verminderte LQ und vermehrt psychosoziale Belastungen. Eine im Jahr 2021 veröffentlichte Metaanalyse von Khaled Ezzedine et al. mit 1799 Patienten bestätigte die Beeinträchtigung der LQ der Patient*innen durch die Erkrankung ebenso wie weitere aktuelle systematische Literaturübersichten [27, 55]. Die Analyse von 130 eingeschlossenen Studien durch Mauro Picardo et al., die zwischen 1996 und 2021 veröffentlicht wurden, verdeutlicht, dass psychosoziale Belastungen nicht nur die Patienten selbst, sondern auch deren Familien und Pflegende betreffen [55]. Nur in wenigen Studien wurden keine Unterschiede in der psychosozialen Belastung gefunden, was von den Autoren mit methodischen Besonderheiten begründet wird [50, 52].

### Wichtige betroffene Problembereiche

**Figure 1:**
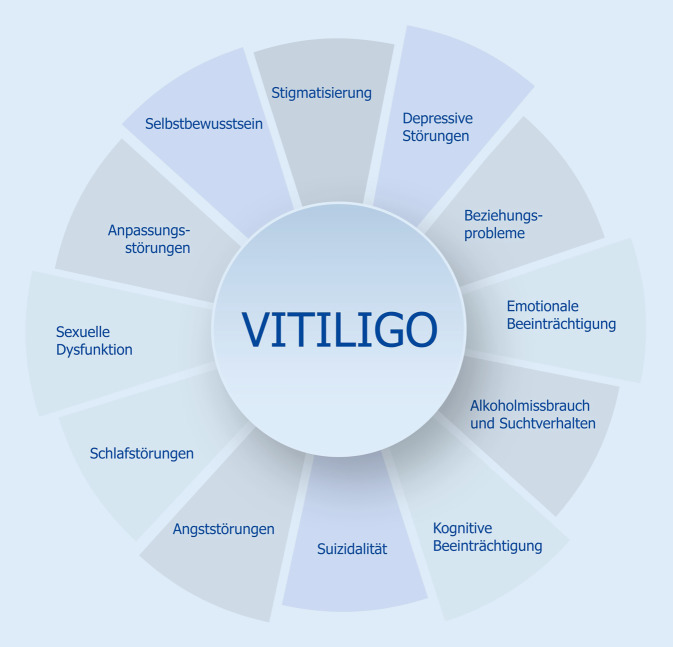

Nationale und internationale Studien haben gezeigt, dass die psychosozialen Belastungen und Einbußen an LQ unterschiedliche Problembereiche der Patienten betreffen können (Abb. 1). Häufig geht Vitiligo mit psychischen Störungen einher, insbesondere mit Angst und Depression [19, 27, 69]. Dabei korreliert das beobachtete Ausmaß der beiden Krankheiten mit der Schwere und Aktivität der Vitiligo [23]. Einer aktuellen systematischen Übersichtsarbeit mit 168 eingeschlossenen Studien zufolge wird am häufigsten über Depression (0,1–62,3 %, 41 Studien) und Angst (1,9–67,9 %, 20 Studien) berichtet. Häufigste psychosoziale Komorbiditäten betreffen das Gefühl der Stigmatisierung (17,3–100 %), Anpassungsstörungen (4–93,9 %), Schlafstörungen (4,6–89,0 %), Beziehungsprobleme (2,0–81,8 %) und Vermeidungsverhalten (12,5–76 %). Zudem kann die Hautkrankheit die sexuelle Funktion von Frauen und Männern erheblich beeinträchtigen [48, 54, 71]. Studien zufolge scheint insbesondere die genitale Lokalisation der Vitiligo einen negativen Einfluss auf die LQ und sexuelle Funktion der betroffenen Frauen und Männer zu haben [65, 71, 76]. Oft bestehen Probleme gleichzeitig in mehreren Bereichen, was zu einem niedrigen Selbstwertgefühl und sozialer Isolation der Betroffenen beiträgt.

Eine besondere Krankheitsbelastung weisen auch Kinder mit Vitiligo auf [17]. Betroffene Kinder sind weniger aktiv und neigen dazu, ihre Läsionen zu verstecken. Bei Eltern von Kindern mit Vitiligo waren vermehrt Symptome einer Panikstörung und depressiver Episoden zu beobachten [66]. Ein Konsensuspapier unterstreicht die hohe Belastung der Lebensqualität auch bei Kindern und betont die Notwendigkeit, zu deren Erfassung kindgerechte, spezifische Verfahren einzusetzen [20].

### Wer ist besonders von psychosozialer Beeinträchtigung gefährdet?

Die Vulnerabilität für eine psychosoziale Belastung durch die Erkrankung ist in verschiedenen Patientengruppen unterschiedlich [4] hoch. Die Studie von Reza Bidaki et al. zeigte, dass (alleinerziehende) Frauen einen besonders starken Leidensdruck erfuhren. Dies lag an mehreren Faktoren. Zum einen waren sowohl Gesichts- als auch Halsregionen von Vitiligo betroffen, zum anderen betraf die Krankheitsdauer weniger als 5 Jahre. Gleichzeitig war die soziale Akzeptanz gegenüber der Sichtbarkeit von Vitiligo signifikant geringer [13]. Auch in weiteren Studien war die Belastung durch Vitiligo bei Frauen besonders stark ausgeprägt [67]. In einer Klinikstudie war das Risiko für psychosoziale Belastung besonders bei jüngeren Frauen im Alter zwischen 20 und 39 Jahren, bei Betroffenen mit höherer Bildung und kurzer Krankheitsdauer sowie bei generalisierter Erkrankung erhöht [75]. Während in einem Teil der Studien die Hautfarbe und Ethnizität keinen oder keinen wesentlichen Einfluss auf die erlebte Belastung durch Vitiligo hatten [37, 56], zeigten sich Menschen mit dunklerer Haut in anderen Studien stärker belastet [29, 44, 56]. Neben der Lokalisation beeinflusst auch die Größe der hypo- oder depigmentierten Läsionen das Ausmaß der psychosozialen Beeinträchtigung [54]. Vor allem ein früher Krankheitsbeginn in der Kindheit oder Jugend wirkt sich negativ auf die spätere LQ aus [54]. Zudem wurde in einer kleineren Studie gezeigt, dass bei Kindern und Jugendlichen mit Vitiligo (*n* = 29) häufiger belastende frühere Lebensereignisse zu verzeichnen sind als bei Kindern und Jugendlichen mit Alopecia areata (*n* = 31) oder nach Alter und Geschlecht gematchten Hautgesunden (*n* = 30) [66].

Schließlich legt eine Querschnittsstudie der französischen „ComPaRe Vitiligo e‑cohorte“ (*n* = 602) nahe, dass wahrgenommener Stress bei vielen Patienten den Ausbruch der Erkrankung triggern oder Symptome und klinische Zeichen verschlimmern kann [21]. Hierbei stellten ein höheres Alter bei Krankheitsbeginn, komorbide Atopie und familiäre Vitiligo die wichtigsten Einflussfaktoren für den wahrgenommenen Stress dar.

### Kumulative Lebensbelastung und Stigmatisierung

Die kumulierenden Belastungen über längere Lebensphasen beinhalten verminderte berufliche Chancen, Beeinträchtigungen im sozialen Bereich wie auch emotional chronifizierte Belastungen bis hin zu manifesten psychischen Erkrankungen. Verpasste Gelegenheiten und Chancen wie Partnerwahl und Heirat sowie berufliche Einschränkungen wurden berichtet. Diese kumulierenden, zum Teil irreversiblen Belastungen werden zusammenfassend als kumulative Lebensbelastung („cumulative life course impairment“ [CLCI]) bezeichnet [5] und wurden für Vitiligo besonders betont [46].

Im Rahmen sozialer Interaktionen erleben Patienten oft Stigmatisierung [18], da die sichtbaren Areale – und im intimen Bereich auch die für die Öffentlichkeit nicht sichtbaren Körperbereiche – als entstellend erlebt werden. Als belastend wird auch eine häufig als unzureichend erlebte Unterstützung durch Ärzte und Familie empfunden [11].

## Begleiterkrankungen bei Vitiligo

Populationsbasierte Untersuchungen fanden bei Vitiligo vermehrt dermatologische Begleiterkrankungen, speziell chronisch entzündliche Hauterkrankungen wie atopische Dermatitis, Psoriasis und Lichen planus und Autoimmunerkrankungen (Alopecia areata) [49]. Des Weiteren haben Menschen mit Vitiligo eine deutlich erhöhte Komorbidität in Form von internistischen Erkrankungen [24], die nach der Erstdiagnostik an der Haut frühzeitig abgeklärt werden können. Beispielhaft genannt seien Schilddrüsenerkrankungen (Thyreoiditis), Diabetes mellitus, perniziöse Anämie, Autoimmunerkrankungen wie das Sjögren-Syndrom und der systemische Lupus erythematodes, entzündliche Darmerkrankungen und zahlreiche weitere Syndrome auch im Augen‑, Hals-Nasen-Ohren-ärztlichen und neurologischen Bereich.

Vitiligo wird oftmals als Erkrankung wahrgenommen, bei der es an UV-Schutz mangelt und die ein erhöhtes Risiko für die Entwicklung von Hautkrebs besitzt. Dies kann bei Dermatologen zu einer nicht-leitliniengerechten Therapie beitragen. Interessanterweise zeigen große epidemiologische Studien jedoch, dass die Rate an NMSC (nichtmelanozytärer Hautkrebs) und Melanom bei Personen mit Vitiligo nicht höher ist als bei solchen ohne diese Pigmenterkrankung [8, 34, 73].

## Krankheitslast und Therapiebedarf

In den meisten Ländern und Regionen weltweit gilt Vitiligo als eine medizinisch relevante und eindeutig behandlungsbedürftige Erkrankung [31, 39, 53, 72]. Das Spektrum möglicher Therapieansätze ist zwar breit, jedoch sprechen Patienten auf die bisher üblichen Therapien meist nur unzureichend an [6, 59]. Ein Großteil der Patienten hat daher einen noch nicht gedeckten Versorgungsbedarf, obwohl eine Therapie gemäß der aktuellen Leitlinie angezeigt wäre. Aktuelle Empfehlungen umfassen als mögliche, wenngleich oft nicht ausreichend wirksame Therapieoptionen insbesondere systemische Immunsuppressiva, topische Kortikoide, Calcineurininhibitoren, Phototherapien, Kombinationen der vorgenannten Maßnahmen, Immunmodulatoren, Hautzelltransplantation und supportive Therapien [1, 15, 33].

Angesichts der bislang äußerst begrenzten Heilungschancen für Vitiligo kommt auch den psychosozialen Behandlungen, z. B. psychotherapeutischen Interventionen etwa mit kognitiver Verhaltenstherapie oder analytischer Psychotherapie, eine hohe Bedeutung zu [15, 25, 62]. Die Indikation für entsprechende Interventionen ist vielfach analysiert und bestätigt worden [3, 62, 72]. Die aktuelle systematische Literaturübersicht bestätigt, dass eine Behandlung der Vitiligo signifikant (*p* < 0,05) zu einer besseren LQ der betroffenen Patienten beitragen kann [55]. Neben medizinischen und psychologischen Therapieansätzen stellt die Anbindung an eine Selbsthilfegruppe einen v. a. für höhergradig betroffene Vitiligopatienten empfehlenswerten Bestandteil einer umfassenden Versorgung dar [70].

Auch in Deutschland fand sich in allen publizierten Studien bei Patienten mit Vitiligo trotz Behandlung mit verfügbaren Therapieoptionen [59] eine klinisch relevante Krankheitsbelastung [58, 68]. Aus dieser unbefriedigenden Situation kann zweifelsfrei der Bedarf einer sachgerechten Therapie einerseits sowie der Entwicklung wirksamer neuer Therapieoptionen andererseits abgeleitet werden [52]. Die in 4/2023 von der EMA (Europäische Arzneimittel-Agentur) zugelassene topische Behandlung mit dem JAK(Januskinae)-1/2-Hemmer Ruxolitinib bei Patienten mit nichtsegmentaler Vitiligo ab dem 12. Lebensjahr stellt hierbei einen ersten Schritt der zielgerichteten Behandlung dar.

Die frühzeitige korrekte Diagnose und langfristige Betreuung der Patienten unter Therapie sind auch deswegen sinnvoll, weil Vitiligo zahlreiche relevante Differenzialdiagnosen aufweist, etwa maligne Tumoren oder Autoimmunerkrankungen der Haut [14].

Für die Indikationsstellung zur topischen oder systemischen Therapie sind replizierbare Kriterien notwendig, wie sie in Form von Checklisten bereits in die Leitlinie zur Therapie der atopischen Dermatitis aufgenommen wurden. Neben der klinischen Charakterisierung ist die Erfassung der Krankheitslast und der Einbuße an Lebensqualität für die Therapieentscheidung bei Vitiligo von besonderer Bedeutung. Im Zuge der Weiterentwicklung der Vitiligo-Leitlinie ist auch ein Konsens zur Erfassung dieser Parameter notwendig. Aus dieser Definition leitet sich dann auch der generelle Versorgungsbedarf von Vitiligo in Deutschland ab.

## Fazit für die Praxis

- Vitiligo ist weltweit eine häufige, autoimmunologisch bedingte Pigmentstörung.
- Sie stellt für einen relevanten Teil der Betroffenen eine ernst zu nehmende Erkrankung mit einem entstellenden, belastenden Charakter dar.
- Die Erkrankung geht regelhaft mit einem ausgeprägten Leidensdruck, signifikanten Einbußen der Lebensqualität und der Notwendigkeit einer fundierten differenzialdiagnostischen Abklärung einher.
- Die Behandlung der Vitiligo orientiert sich sowohl an medizinischen wie auch psychosozialen Zielen und dient der Linderung oder Vermeidung einer kumulierenden, chronischen Lebensbelastung.
